# Applying multilevel selection to understand cancer evolution and progression

**DOI:** 10.1371/journal.pbio.3003290

**Published:** 2025-07-18

**Authors:** Lucie Laplane, Anaïs Lamoureux, Harley I. Richker, Gissel Marquez Alcaraz, Angelo Fortunato, Zachary Shaffer, Athena Aktipis, Paul S. Mischel, Anya Plutynski, Jeffrey P. Townsend, Carlo C. Maley

**Affiliations:** 1 UMR8590, CNRS & University Paris I Pantheon Sorbonne, Paris, France; 2 UMR1270, Inserm & Gustave Roussy Cancer Campus, Villejuif, France; 3 Arizona Cancer Evolution Center, Arizona State University, Tempe, Arizona, United States of America; 4 Center for Evolution and Medicine, Arizona State University, Tempe, Arizona, United States of America; 5 Biodesign Center for Biocomputing, Security and Society, Arizona State University, Tempe, Arizona, United States of America; 6 School of Life Sciences, Arizona State University, Tempe, Arizona, United States of America; 7 Department of Theoretical and Applied Sciences, eCampus University, Novedrate, Italy; 8 Department of Psychology, Arizona State University, Tempe, Arizona, United States of America; 9 Global Futures Lab, Arizona State University, Tempe, Arizona, United States of America; 10 Department of Pathology, Stanford University School of Medicine, Stanford, California, United States of America; 11 Sarafan ChEM-H, Stanford University, Stanford, California, United States of America; 12 Department of Philosophy, Affiliate with Division of Biological and Biomedical Sciences, Washington University in St Louis, St Louis, Missouri, United States of America; 13 Department of Biostatistics, Yale School of Public Health, New Haven, Connecticut, United States of America; 14 Department of Ecology and Evolutionary Biology, Yale University, New Haven, Connecticut, United States of America; 15 Program in Computational Biology and Biomedical Informatics, Yale University, New Haven, Connecticut, United States of America; 16 Program in Genomics, Genetics, and Epigenetics, Yale Cancer Center, New Haven, Connecticut, United States of America; Princeton University, UNITED STATES OF AMERICA

## Abstract

Natural selection occurs at multiple levels of organization in cancer. At an organismal level, natural selection has led to the evolution of diverse tumor suppression mechanisms, while at a cellular level, it favors traits that promote cellular proliferation, survival and cancer. Natural selection also occurs at a subcellular level, among collections of cells and even among collections of organisms; selection at these levels could influence the evolution of cancer and cancer suppression mechanisms, affecting cancer risk and treatment strategies. There may also be cancer-like processes happening at different levels of organization, in which uncontrolled proliferation at lower levels may disrupt a higher level of organization. This Essay examines how selection operates across levels, highlighting how we might leverage this understanding to improve cancer research, prevention and treatment.

## Abstract 2

Cancer is a prime example of multilevel selection. At an organismal level, natural selection has led to the evolution of diverse tumor suppression mechanisms, while at a cellular level, it favors traits that promote cellular proliferation, survival and cancer. This Essay examines how selection operates across levels, highlighting how we might leverage this understanding to improve cancer research, prevention and treatment.

## Introduction

Natural selection occurs in any population of entities that exhibit heritable variation in their survival and reproductive success [1]. Limited resources amplify the effects of this variation in the “struggle for existence”, although it is not required for natural selection [1]. Evolutionary biologists have long recognized that natural selection can occur across a range of levels of organization, a phenomenon known as multilevel selection [2–4]. Selection at different levels of organization may act independently or they may interact. When they interact, the resulting adaptations can be understood as the combined action of natural selection at different levels, which may be aligned (synergistically selecting for an adaptation) or be in conflict, in which case the resulting character will be some form of compromise between the selective pressures [5,6]. For levels of selection to interact, the results of that selection at both levels must be passed on to the next generation. Cancer is an interesting example of natural selection at the level of organisms that favors organisms with traits that enable them to survive and reproduce before succumbing to cancer, leading to the evolution of cancer defenses. However, cancers are the result of natural selection at the level of the cell, favoring mutations that generate all the hallmarks of cancer [7]. Yet, because the cancerous cells are not generally passed on to the next generation of organisms, the phenotype of cancer susceptibility is only determined by selection at the level of the organism. In other words, natural selection at the level of cells is independent of natural selection at the level of organisms, with respect to cancer susceptibility phenotypes.

**Fig 1:**
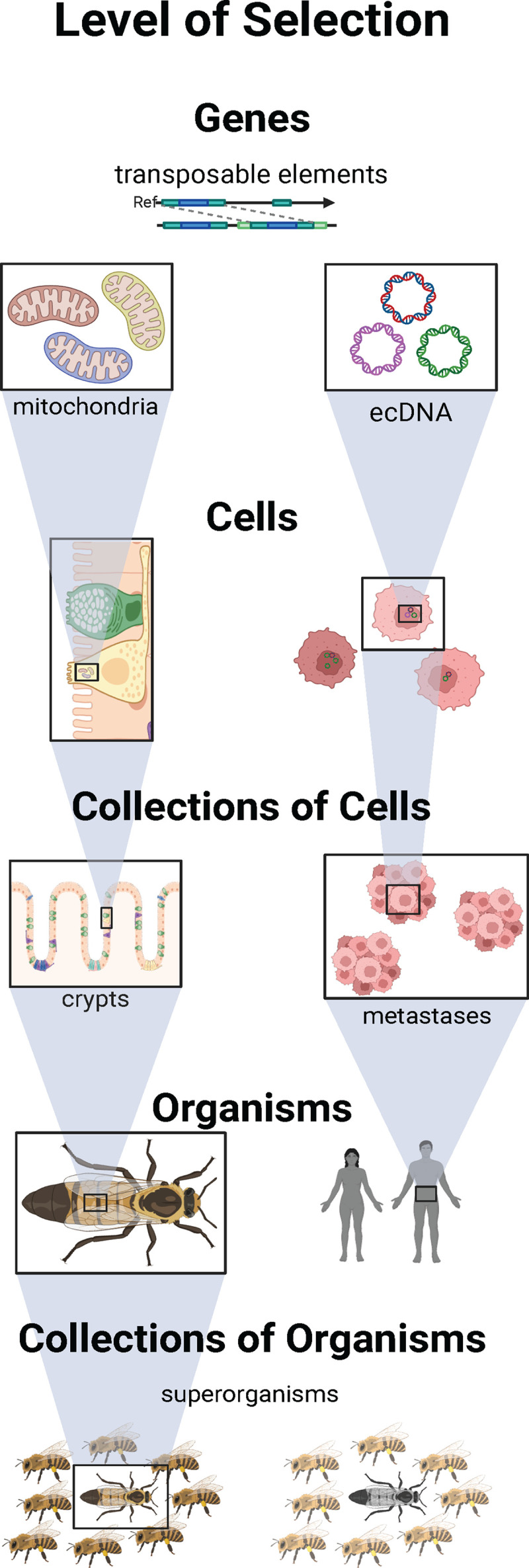
Levels of selection. There may be selection at the level of genes, or collections of genes in the form of transposable elements like retrotransposons, mitochondrial genomes, and extrachromosomal DNA (ecDNA). There may also be selection at the level of cells, collections of cells (in the form of epithelial proliferative units such as intestinal crypts or metastases), organisms or even collections of organisms in the form of eusocial superorganisms. Figure created with BioRender, https://BioRender.com.

The focus of most cancer research has been on the results of (independent) natural selection at the levels of cells and organisms. In this Essay, we show that natural selection also acts on genes, collections of cells and collections of organisms (Fig 1). Selection at these other levels can influence cancer risk, either promoting or inhibiting progression, with the possibility of also impacting cancer treatment strategies. Multilevel selection can also generate cancer-like phenomena if entities at any level start reproducing out of control to the point that they compromise a higher level of organization. This may select for cancer suppression mechanisms at multiple levels of organization as well. Here, we examine how selection operates across levels and identify important opportunities for leveraging this understanding to improve cancer research, prevention, and treatment. For each level of organization, we note whether conditions of natural selection have been observed: heritable variation that affects the survival and reproductive success of entities at that level (Table 1). At the end, we analyze which of these levels of selection interact and which are independent.

**Table 1:** Which levels of selection meet the criteria for natural selection.

Level of selection	Variation	Variation is heritable	Intrinsic fitness differences
Cells	✓	✓	✓
Extra-chromosomal DNA	✓	✓	?
Retrotransposons	✓	✓	?
Mitochondria	✓	✓	✓
Epithelial proliferative units	✓	✓	?
Cancer stem cells and their progeny	✓	✓	?
Metastases	✓	?	?
Organisms	✓	✓	✓
Superorganisms	✓	✓	✓

## Cells as units of selection

Individual cells in normal tissues and tumors acquire somatic genetic mutations [8]. Many of these mutations are neutral and do not substantially affect cell fitness [9] (defined as the ability of individual cells to survive and reproduce). However, some somatic genetic mutations confer a selective advantage that enables the mutant cells to survive, proliferate and outcompete other cells within the tumor microenvironment [10–13]. Therefore, at a very basic level, cancer is an outcome of cellular competition [14], and the cell is a fundamental unit of selection operating in the development of cancer (Table 1). This competition is context-dependent, influenced by factors such as the availability of limiting resources such as space, nutrients and oxygen, which vary substantially between normal and tumor tissues. For example, in normal epithelial tissues, cells are organized in two-dimensional sheets, and a mutant cell may only realize a proliferative advantage if space becomes available or cells adapt to grow in three dimensions [15]. The nature of this competition likely changes during neoplastic progression as distinct resources become limiting, and depending on whether competition is primarily about rate of reproduction, survival or resource acquisition (including space) [16].

Variation among cells is generated by a number of mechanisms during a person’s lifetime, owing to various factors such as errors in DNA replication, errors in replication of epigenetic modifications during DNA synthesis, defects in DNA repair mechanisms or exposure to mutagens. At the most basic level, any replicating system will occasionally make mistakes, so some mutational processes are endogenous and cannot be prevented by avoiding mutagens. Other mutational processes are exogenous; meaning exposure can be prevented. Classic epidemiological and laboratory studies have led to estimates that 40% of the human cancer burden is explained by known risk factors such as tobacco and obesity, raising the question of what is driving the rest of the cancer burden [17]. Recent studies on the mutagenic basis of cancer show that the ratio of endogenous, unavoidable mutations to exogenous, presumably preventable mutations ranges widely depending on the cancer type, from melanomas that are >80% driven by UV light to gliomas that are >95% driven by endogenous processes [18].

Whether caused by endogenous or exogenous processes, somatic gene mutations and epigenetic alterations can alter the fitness of cells by altering cellular functions that enhance cellular survival or reproduction. These (epi)mutations can affect critical genes involved in cell growth, division, death and other cellular processes, playing crucial roles in the development of cancer. For instance, the driving amino acid substitutions conferring the greatest selective benefit to individual cells in lung adenocarcinoma include the mitogen-activated protein kinase pathway genes *EGFR*, *KRAS*, and *BRAF*, as well as the tumor suppressor *TP53* [12]. The driving amino-acid substitutions that are most beneficial for lung squamous-cell carcinoma cells occur in *PIK3CA*, which regulates cell growth and survival, and *TP53*, which regulates a variety of functions including cell cycle and apoptosis.

However, the fitness benefit of a somatic genetic change can depend not just on the tumor microenvironment, but also on previous somatic genetic changes [10,19]. Moreover, these gene interactions can potentially impact cancer therapies and patient outcomes [20]. The conditionality of selective benefit of mutations is referred to as selective epistasis: the effect on selection of the direct or indirect interaction between different genetic mutations [21]. In cancer cells, epistatic interactions can occur when the effects of one mutation depend on the presence or absence of other mutations [22]. For example, if *TP53* is intact, many mutation events are deleterious because they trigger *TP53*-mediated apoptosis. But if *TP53* is inactivated by mutations, massive genomic instability can ensue. This interplay between mutations can influence the overall fitness of the cell and its ability to survive and proliferate.

The presence of somatic mutations alone—even in canonical cancer driver genes or their combinations—does not inevitably result in tumor formation [23,24]. First, somatic mutations can sometimes play a role in the prevention of cancer rather than contribute to its development. Two examples of genes in which some mutations may contribute to clonal expansions while suppressing progression to cancer include *KLF4* in intraductal papillary mucinous neoplasms and *NOTCH1* in esophageal cancer [25–28]. Moreover, mutant but not yet malignant cells may outcompete mutant and already malignant cells [27,29,30]. Second, somatic mutations must occur in a particular cell type to form a tumor. The same set of mutations may lead to cancers with different properties depending on the cell of origin (i.e., the type of cell that is mutated) [31,32]. Somatic mutations must also encounter tumor-promoting tissue conditions that tip the balance from controlled to uncontrolled growth [23,33]. These tumor-promoting conditions constitute a complementary, physiological component to the mutagenic causation of cancer [34]. Tumor-promoting or tumor-suppressive conditions such as exposure to physiological toxins, the cumulative effects of exercise and aging, as well as the specific stage of cancer development each alter the selection on individual somatic mutations [34–36], playing key roles in the fate of tumors and of those who are afflicted with them.

A consequence of both somatic genetic selective epistasis and the evolving tumor microenvironment is stage-specific selection: mutations may have distinct fitness effects on cancer cells at distinct stages of cancer progression [28,37]. For example, mutations that promote rapid cell division may be advantageous during the early stages of tumor growth, while mutations that allow cells to invade surrounding tissues or evade immune surveillance may become advantageous at later stages. The availability of specific resources such as space [15,38,39], nutrients [40] and oxygen [41] varies significantly across tumorigenesis, metastasis and therapy resistance [42,43], influencing which mutations provide a selective advantage. Stage-specific selection acts as a driving force shaping the evolutionary dynamics of cancer cells and determining their behavior at serial stages of disease progression, including during therapy [44]. A key aspect of how a tumor evolves through time is its interactions with the tumor immune microenvironment. Thus, it will be important not only to contextualize estimates of the selective impact of mutations within their specific somatic genetic landscape, but also to do so within the broader microenvironmental context—including immune context—that modulates tumor growth and survival.

In summary, focusing on cells as units of selection in cancer highlights the role of somatic mutations, natural selection, epistasis, stage-specific selection and evolutionary trajectories in driving the growth, progression and heterogeneity of tumors. Viewing cancer through an evolutionary lens provides insights into the dynamic nature of the disease and informs strategies for its diagnosis, treatment and management. For example, we should be asking how we can change the microenvironments of pre-cancers and cancers to select for desired phenotypes. We might also measure the rate of evolution in neoplastic cells as prognostic or predictive biomarkers, and as targets for cancer prevention efforts based on slowing down the rate of neoplastic progression [45].

## Genes as units of selection

In much of evolutionary biology, it has been helpful to consider natural selection primarily at the level of the gene [46]. This predominance arises because sexual recombination and horizontal gene transfer enable genes to be reshuffled among other genes with which they must interact. Consequently, the average fitness effect of a gene is integrated over the backgrounds of all collaborating genes within associated populations. In cancer cells, the conventional wisdom has been that there is little to no recombination or horizontal gene transfer, though it is possible that cell fusions and exchanges of cellular contents through nanotubes or extracellular vesicles may provide some degree of gene-level selection [47,48]. In addition, there are at least two other phenomena enabling selection at the level of genes instead of genomes: extra-chromosomal DNA (ecDNA) and retrotransposons. There may also be selection of collections of genes that are independent of the nuclear chromosomes, in the form of mitochondrial genomes.

### Extra-chromosomal DNA

Circular bits of DNA were first observed in cancers back in 1962 when they were called double minutes [49]. These ecDNAs lack centromeres and are randomly segregated during cell division [50], leading to rapid changes in copy numbers of ecDNA over cell divisions. ecDNA particles are enriched for oncogenes, regulatory elements and immunomodulatory genes [51], often at high copy numbers (e.g., in the hundreds [52]).

The enrichment of the number of ecDNA particles suggests that selection may be happening at the level of ecDNAs. For that selection to occur, there must be variation among the ecDNAs, and that variation must affect their ability to replicate and survive. There is clear evidence of variation among ecDNAs in a tumor [53–55]. There is also evidence for continuing generation of further variation in the ecDNAs over time [51,56]. It is hypothesized that this variation enhances the likelihood that structural variants or sequence changes that enhance cell fitness will occur and be selected at the cellular level [57]. The replication of ecDNA was thought to be tied to the cell cycle: each ecDNA only replicates in the phase when normal cells conduct ordinary DNA synthesis [58]. However, recent evidence suggests that this may be frequently dysregulated and highly variable [59]. There is also evidence that smaller ecDNAs have a survival advantage because they are less likely to be extruded from the cell in micronuclei [60], suggesting selection at the ecDNA level. Furthermore, ecDNAs evolve, accumulating structural complexity over time within a tumor [61] and acquiring persistent point mutations [56]. It is just not clear if ecDNAs are evolving by natural selection at the gene level or by other mechanisms (such as genetic drift or selection at the cellular level; Table 1).

There is clear evidence of alignment of selective pressures on ecDNAs and cancer cells. ecDNAs carry genes that benefit cancer cells [50,51], and patients that have cancers with ecDNAs have a far worse prognosis than patients with other types of gene amplifications or those whose tumors lack oncogene amplifications [51]. ecDNA can also arise in pre-cancers during neoplastic progression. When Barrett’s esophagus pre-cancerous tissue has ecDNAs, it is extremely likely to evolve into fully-fledged esophageal cancer [61]. Prescient work by Robert Schimke and others [62] showed that these ecDNA elements could drive resistance to methotrexate by amplifying the dihydrofolate reductase gene, suggesting an adaptive form of gene amplification.

Prior to the demonstration that ecDNA plays a role in dynamic resistance of tumors to targeted therapy [63], ecDNA was thought to be rare (1.4% of tumors according to the Mitelman database of chromosomal aberrations) and of unknown importance. An extensive series of studies has now demonstrated that ecDNA is common, particularly in solid cancers, and affects both children and adults. A recent study of nearly 15,000 tissue samples of 39 different tumor types revealed ecDNA in 17.1% of samples, with an increased frequency in those at an advanced tumor stage and in metastases [51]. It has not yet been shown whether those increased frequencies are necessarily linked to selection as opposed to being a secondary effect of degraded genetic instability with oncogenic progression.

### Retrotransposons

Retrotransposons are mobile DNA sequences that reproduce and move within the genome using a ‘copy and paste’ mechanism. This process enables retrotransposons to replicate independently of the cell cycle. They are transcribed into RNA then reverse-transcribed back into DNA, which integrates the offspring retrotransposon at a new genomic location while the parental retrotransposon remains at its original place. Such replication can lead to evolution at the retrotransposon level, if some retrotransposons replicate more than others.

Gene-level selection and evolution of retrotransposons is unlikely to occur in normal human cells, where retrotransposons are actively repressed with few exceptions (e.g., specific retrotransposons expressed at specific developmental stages) [64,65]. This repression is maintained through epigenetic mechanisms such as DNA methylation and histone modifications that promote heterochromatin formation and transcriptional silencing [66,67]. However, in cancers, retrotransposons are often reactivated. Active retrotransposition can be observed within cells of many cancers [68,69], a process associated with DNA hypomethylation and disrupted histone regulation [68]. One multi-sample study of pancreatic ductal adenocarcinoma comparing primary tumors and metastases showed that insertions of the LINE-1 retrotransposon across samples from a single patient were only partially overlapping, and the number of insertions was higher in metastases than in the primary tumor, indicating an increased reproductive success of some LINE-1 in some cancer cell lineages [70]. But whether this differential fitness is intrinsic to LINE-1 is unclear. It could only reflect variations in the epigenetic state of the cells, providing more or less opportunities for different LINE-1s to replicate in different cells. The fact that Alu elements derive from LINE-1s and yet outnumber them in the human genome suggests that Alus evolved a higher fitness than LINE-1s, possibly by reducing their size and parasitizing the retrotransposition genes of LINE-1s [71].

In addition to these potential examples of positive selection, negative selection has clearly been at work. Genomes are littered with a large number of retrotransposons that are no longer able to copy and paste due to mutations. It remains undocumented whether negative selection acts on retrotransposons during cancer progression, but it is quite possible that sufficient data to study this is already available (Table 1). Proving that retrotransposons are negatively selected for during cancer progression would require careful phylogenetic reconstruction of retrotransposons in multi-region, longitudinal and/or single-cell sequencing studies.

### Mitochondria and mitochondrial genomes

Like ecDNA and retrotransposons, mitochondria can replicate independently of cell replication [72]. They also accumulate alterations that they transmit to their descendants when they divide. They can therefore evolve, at least through neutral evolution. Whether they evolve by natural selection depends on whether their reproductive success is affected by heritable properties of the mitochondrial genome. The phenotypes of mitochondria and the replication of their genomes are largely regulated by genes in the cell nucleus and by the energy demands placed on the cell. For example, muscle cells vary greatly in mitochondrial content with organismal physical activity [73]. Given the nucleus’ dominant role in mitochondrial reproduction, it might seem impossible for a mitochondrial variant to have a reproductive or survival advantage over other mitochondria.

It is easier to track the success of mitochondrial genomes rather than mitochondria, because mitochondria are constantly fusing and dividing. In addition, mitochondria often contain multiple mitochondrial genomes. However, if we ignore the fluid packaging of mitochondrial genomes, there are clear instances where mutations in the mitochondrial genome are deleterious for that genome, triggering mitochondrial death due to mitophagy [74,75]. There are also clear instances where a variant mitochondrial genome that is initially in the minority takes over the population of mitochondrial genomes in a cell [76–78]. This selection at the scale of mitochondrial DNA (mtDNA) leads these variants to be referred to as “selfish mtDNA” [79] (Table 1). Known examples include mtDNA with deletions that make them replicate faster than wild type mtDNA [79], mtDNA mutations that recruit replication machinery, enhancing their reproduction [80,81], and epigenetic (and heritable) alterations that bias mtDNA toward replication and away from transcription [82–84].

Selfish mutations in mtDNA that interfere with their function in the cell are often countered by selection at the cellular level. However, there is evidence of horizontal transfer of mitochondria between cells, through tunneling nanotubes [85,86]. Moshoi and colleagues [87] demonstrated that more than 10% of the mitochondrial mass may be transferred this way, opening a potential channel for selfish mtDNA to escape their host cells before cell level selection removes them. This transfer of mitochondria may, however, be an adaptation at the cellular level, rather than a benefit to the mitochondria. The formation of tunneling nanotubes is increased after chemotherapy or oxidative stress [85,88] and appears to function in a way that enables cancer cells with damaged mitochondria to acquire healthy mitochondria from normal cells, rescuing their essential aerobic function [86,89–91].

## Collections of cells as units of selection

Natural selection can act on groups of cells and not just individual cells. The concept of group selection is typically discussed in the context of organisms, but it can also apply to cellular groups.

Damuth and Heisler [3,89] distinguished two types of group selection. In the case of multilevel selection 1 (MLS1), the fitness of the entities that compose the group is affected by group membership. In the case of multilevel selection 2 (MLS2), fitness is attributed to the group itself and depends on the properties of the collective group. In MLS2, group-level properties, such as the division of labor in social organisms, might lead some groups to survive longer or reproduce more frequently, generating more groups carrying those properties (Table 2). Some have argued that group selection processes of MLS1 could evolve into MLS2 [3,92].

**Table 2:** Is multilevel selection of type 1 (MLS1) and/or of type 2 (MLS2)?

Level of selection	MLS1	MLS2
Epithelial proliferative units	?	✓
Cancer stem cells and their progeny	?	?
Metastases	✓	?

In the following section, we examine three types of groups of cells that can undergo selection: stem cells and their progeny (including proliferative units in an epithelial tissue), a cancer stem cell with its non-stem cell progeny in a neoplasm, and metastases.

### Epithelial proliferative units

In many epithelial tissues, where the majority of cancers arise [93], cells are organized into proliferative units. These are called crypts in the stomach, intestine and Barrett’s esophagus; acini in breast, lung, salivary gland, pancreas and liver tissue; epithelial proliferative units in the skin; and simply glands in the endometrium and prostate. Cairns pointed out in 1975 that the architectural constraints of subdividing a tissue into isolated small sets of stem cells and their progeny (glands), can itself act as a tumor suppressor [94]. A mutant stem cell with a cell-level selective advantage may take over the stem cell population of a gland, but there is no clear way it could expand into neighboring glands. In many epithelial glands, non-stem cells differentiate and are ejected from the tissue in a matter of days [95]. If a mutation occurs in a non-stem cell, it will soon be removed from the tissue, unless the mutation interferes with the differentiation process itself.

Epithelial proliferative units can divide [96] (and fuse [97,98]), die [99] and mutate [96], making them a potential substrate for natural selection (MLS2, as those are properties attributed to the group; Tables 1 and 2). Early clonal expansions in tissues are thought to occur in part through the division of epithelial proliferative units. Natural selection at the proliferative unit level should select for mutations that trigger proliferative unit division and select against mutations that lead to the death of proliferative units. There is evidence that mutations in *CDKN2A* cause clonal expansion in the crypt structured epithelium of Barrett’s esophagus, but have little effect on the risk of progressing to esophageal cancer [100]. This indicates that cellular level selection for those mutations may not be part of the evolutionary trajectory that leads to esophageal cancer. Similarly, there is evidence that *NOTCH1* mutations drive clonal expansions in skin [101] and squamous esophageal tissue [102], but may even protect against the evolution of cancer [102]. Furthermore, colorectal cancer is thought to start with clusters of aberrant crypts, called aberrant crypt foci, which eventually evolve into polyps.

If proliferative units are also units of selection, then the same logic that leads to the evolution of cells dividing out of control should also lead to proliferative units dividing out of control, which would generate a mass of proliferative units. This is what a colonic adenoma looks like. In fact, we propose that many ‘well-differentiated tumors’ (tumors that have retained much of the differentiation structure of the tissue in which they originated) may actually be generated by selection at the level of the proliferative unit. By contrast, poorly differentiated tumors are likely generated by selection at the level of the cell. Although, there is a continuum between tumors maintaining structured proliferative units and those in which such proliferative units are no longer visible, this difference might help explain why well-differentiated tumors have a better prognosis than poorly differentiated tumors. The rate of evolution depends in part on the population size of the evolving entities. For a well-differentiated tumor of a given size, the number of proliferative units is much less than the number of cells in a poorly differentiated tumor, so well-differentiated tumors should evolve more slowly than poorly differentiated tumors. Loss of differentiation may not only be a hallmark of cancer [103,104], it may also be the reversal of a major transition in evolution, when selection drops down from the proliferative unit level to the cellular level. Another reason that these well-differentiated tumors have better prognosis may be that they maintain much of the normal cell–cell regulatory processes with their neighbors that limit growth and reduce the likelihood of aberrant cellular behavior. Notably, in tumors in which proliferative units are no longer visible, there might still be a different type of group selection occurring on cancer stem cells and their progeny, which we discuss in the next section.

Unfortunately, relatively little is known about the biology of what causes a proliferative unit to divide (in contrast with extensive knowledge about cell division inside the proliferative unit). Does an increase in the number of stem cells in the proliferative unit trigger division? Or is there some other control mechanism that triggers division and which entails expansion of the stem cell population either before or after division of the gland? Organization of epithelial tissues into proliferative units gives evolution at the organismal level a new set of levers and knobs to control that system, and potentially develop new cancer suppression mechanisms at the proliferative unit level.

One of the interesting implications of this is that the birth and death dynamics of proliferative units (as opposed to the cells within them) may be an as yet unexplored regulatory system that could be targeted to help limit cancer progression. For example, there may be tumor suppressor genes (and oncogenes) that are involved in controlling when a proliferative unit divides and dies (and fuses [98]). They would have an impact on clonal expansions of mutations within tissues, the formation of well-differentiated tumors and the risk of progression to cancer. If such proliferative-unit-level cancer suppression mechanisms exist, they could be leveraged for cancer prevention and treatment.

### Cancer stem cells and their progeny

Not all cells within a neoplasm are evolutionary equal. There is evidence that some cells in a neoplasm act like stem cells, with indefinite replication, whereas other cells have limited replicative potential and thus are evolutionary dead ends at the cellular level of natural selection [94,105]. This structure may be retained from the epithelial proliferative units or other differentiation structures in which the cancers evolved (which are generally niche dependent), or neoplasms may independently co-opt differentiation mechanisms in the cell. In fact, the very existence of cancer non-stem cells is an evolutionary conundrum [106]. At the cellular level of selection, any cancer stem cell that wastes its reproductive potential on generating non-stem cells should have a selective disadvantage compared to cancer stem cells that only divide symmetrically, exclusively producing daughter cancer stem cells [106]. It is possible that the distinction between cancer stem cells and cancer non-stem cells is a holdover from the organizational structure of the normal tissues from which cancers evolve, and there just has not been enough time for natural selection to favor clones of pure cancer stem cells. This view would predict that well-differentiated tumors would have a lower frequency of cancer stem cells than poorly differentiated tumors, and thus evolve more slowly. Another explanation for the presence of cancer non-stem cells is that cancer stem cells benefit from belonging to groups that contain cancer non-stem cells [106]. This would be a case of MLS1 (Table 2). Furthermore, according to the cancer stem cell model, cancer stem cells produce cancer non-stem cells, such that cancer stem cells are considered the units of selection, and cancer non-stem cells as evolutionary dead-ends [107]. As they form lineages, each lineage coming from a cancer stem cell could be considered like a proliferative unit. These proliferative units would be analogous to multicellular organisms, where the germline benefits from the production and action of somatic cells. If this hypothesis is correct, then cancer non-stem cells are maintained in neoplasms because clusters of cancer stem cells and the non-stem cells they produced are acting like proto-multicellular colonies, with the cancer stem cells acting as the germline and the cancer non-stem cells acting as their soma [106]. The fact that cancer stem cells and their non-stem cell progeny are almost genetically identical would facilitate the evolution of such cooperation through kin selection. There can clearly be variation between those colonies of cancer stem cells and their progeny due to heritable differences between cancer stem cells (Table 1). It seems likely, but it is not yet clear, if those differences allow some of those colonies to reproduce or survive better than other colonies, which would be a case of MLS2 (Table 2). In cancers in which the cancer stem cell model does not hold true, especially if any cancer cell can produce any cancer cell, the case for MLS2 is more difficult to make, and the notion of a group that would serve as reproductive units upon which selection can act becomes fuzzy [108].

### Metastases

There may also be natural selection at the level of metastases [109]. Natural selection at the ‘metastasis level’ could take the form of either MLS1 or MLS2 (Table 2). In the case of MLS1, the fitness of the cells in a population of metastatic cells may be affected by membership in a group. In the case of MLS2, the metastatic population itself may be said to bear heritable variation and reproduce, and that heritable variation can in theory affect the ability of the metastases to survive and propagate new metastases.

What is the evidence for either MLS1 or MLS2? A classic experiment from Fidler and Kripke showed that the ability to metastasize can evolve [110]. They injected a melanoma cell line into mice and showed that the variation in the ability to seed metastases was heritable when they derived new cell lines from those metastases and injected them into new mice. This should not be a surprise in that the phenotypes necessary for metastasis (migration, generation of growth signals, generation of angiogenic signals, etc.) are all at least partly genetically and epigenetically encoded. Thus, they may mutate and be passed down to offspring metastases (Table 1).

Whether there is competition between metastases for limited resources is an open question; of note, there need not be competition between metastases to get natural selection, all you need is intrinsic variation in their ability to spawn new metastases. Natural selection does not require competition for limited resources. Metastases that evolve more effective mechanisms for spawning new metastases will tend to spread in the body more than metastases that do not.

One source of evidence for MLS2 is that metastases tend to be generated by clusters of cells, rather than by single cells [111,112]. These clusters seem to be organized with ‘leader’ and ‘follower’ cells [113], with various roles attributed to them depending on migratory modalities. For example, a type of collective migration in colorectal cancer has been described that is similar to ameboid migration, in which the cells at the back propel the cluster [114], contrasting with other forms of cluster migration where the front “leader” cells pull the cluster through focal adhesion. Presumably some such clusters are more effective at propagating than others. Perhaps it is easier to metastasize when cells bring a bit of their microenvironment with them (MSL1) [115]. Metastases that are good at sending out propagules (which may be clusters of cells) and growing in new environments will tend to spread. It may be difficult to discriminate between MLS1 and MLS2 in this context. Because there is an alignment of selection at the cell and metastasis levels, it will be difficult to distinguish the relative contribution of selection at each level to the phenomenon of metastasis.

Although metastases may meet the conditions for evolution by natural selection, their importance is an open question. Two important aspects are whether there are enough generations of metastases to generate significant adaptations at the metastasis level, and whether there is enough heritability between generations of metastases to allow natural selection to generate adaptations at the metastasis level [116].

The amount of selection at the level of metastases is currently unknown, in part because micrometastases are so difficult to observe. If there are only a few generations of metastases, then MLS2 cannot really build on metastatic phenotypes. However, it is possible that there may be far more micrometastases and generations of micrometastases than has been observed. This process would be extremely difficult to observe or measure, and would leave little evidence, especially if some of these micrometastases were small and short lived [109]. Evolution occurring at this level could be an important driver of disease progression and patient mortality, selecting micrometastases capable of generating new metastatic propagules and creating the opportunities for cancer cell collectives to evolve more effective cooperation strategies within them that allow them to most effectively exploit the host and colonize new niches in the body. If selection is indeed operating among metastases, this might be key to understanding the challenges associated with managing late-stage cancer and developing effective treatments and prevention strategies [109].

However, there are still many unknowns about metastases that affect the possibility that natural selection is operating among them. For example, the degree of heritability between parent and offspring metastases is largely unknown. The establishment of every new metastasis is a bottleneck event, where only a small proportion of the originating cell population gets into the next metastasis. This suggests that selection at the level of metastases may go hand in hand with genetic drift (i.e., founder effects) at the cell level, which could enhance differences between metastases. Moreover, if there is so much selection at the level of cells during the establishment and growth of a new metastasis, in a new tissue, the offspring metastasis may bear little resemblance to its parent metastasis, and the characteristics of the parent metastasis that allowed it to survive and reproduce may not be faithfully replicated in its offspring. This would limit the ability of natural selection to act at the level of metastases.

Selection at the level of metastases does make at least one testable prediction: if we map out the lineage of relationships between the metastases and the primary tumor, selection at the level of metastases should generally result in metastases begetting metastases begetting metastases, such that they would typically form a single branch (clade) in the cell lineage. If there is little to no evolution at the metastasis level, then most metastases would be spawned directly from the primary tumor and would form independent branches on the cell lineage. Experimental evidence has tended to show that metastases cluster together on a single branch of the cancer cell lineage [117,118]. Metastasis level selection should also lead to the evolution of metastases that are either more and more independent of the tissues in which they land (e.g., generating their own growth signals), or more and more flexible at adjusting to and flourishing in those new environments. The fact that it is generally easier to derive a cancer cell line from a metastasis than from a primary tumor might be a sign of such a gain of independence from the environment.

## Organisms as units of selection

Organisms can vary in the degree of their cancer defenses and susceptibility, much of which is encoded in their tumor suppressor genes, proto-oncogenes, tissue organization and immune systems. That variation is clearly heritable (Table 1), but does it have an effect on an organism’s fitness? Cancer is generally a lethal disease if left unchecked. So it is reasonable to suspect that cancer susceptibility has a strong effect on fitness.

It is tempting to dismiss cancer as a selective pressure on organisms because cancer is generally a disease of old age, after reproduction in most humans [119]. But this perspective is mistaken in three ways. First, humans have a positive impact on the fitness of their children, long after their children are born, leading to the potential for inclusive fitness effects to favor cancer suppression in old age [120]. There is a long period of parental investment that even extends to grandchildren; for example, maternal grandmothers increase the fitness of their grandchildren [121]. Second, childhood cancers and early onset cancers kill people long before they complete their reproduction. In the United States from 2017 to 2019, 5.9% of women and 3.5% of men developed cancer before age 50 [119]. It does not take many such deaths for a disease to exert selective effects on a large population. Third, the reason cancer primarily occurs in old age is thought to be that cancer was a selective pressure on our ancestors, removing individuals from the population that were susceptible to early onset cancers before they could reproduce much. Thus, there is, and has been, natural selection at the level of organisms on their ability to suppress cancer. This can be seen most dramatically in the origin of many tumor suppressor genes at the point when multicellular life evolved [122].

The degree to which cancer has been a selective force in the evolution of organisms depends on their ecology. If there have been other sources of mortality that kill organisms long before they develop cancer (e.g., infectious disease, predators, etc.), then natural selection would have generated adaptations to avoid or suppress those sources of mortality. In some cases, there may be tradeoffs between cancer defenses and other defenses [123]. For example, a prey species might evolve a high metabolism that helps it out-run predators, even if that metabolism leads to more oxidative stress, somatic mutations and eventually cancer. There has clearly been selection on organisms for wound healing, including angiogenesis, but those wound healing pathways are a vulnerability that cancers often exploit [124].

There may also be tradeoffs between cancer susceptibility and other aspects of fitness, such as reproduction [125–127]. There is some evidence that *BRCA1*/*BRCA2* mutations, infamous for increasing the risk of cancer, are also associated with greater fertility [128]. There is also evidence that polymorphisms in *TP53* are associated with both higher fertility [129,130] (including twinning [131]) and higher cancer risk [132–134].

The fact that the ecology of a species has implications for the degree to which cancer was a selective pressure on its evolution has an intriguing implication. If we can identify species that have been under strong selection from cancer mortality, they should have evolved effective cancer suppression mechanisms. We may learn from those species methods to improve cancer prevention in humans [135].

## Collections of organisms as units of selection

Cancer literature regularly borrows concepts from social evolution theories. For example, describing cancer cells as ‘selfish’ or ‘cheaters’, terms normally used in eusocial societies, and describing the anti-cancer mechanisms of organisms as ‘policing’ mechanisms in these societies [123,136–138].

Eusocial societies (with reproductive division of labor and overlapping generations) and especially ‘superorganismic’ ones (including leaf cutter ants, honeybees and others) are often discussed as paradigmatic cases of selection at multiple levels and especially at the level of the group [139] (Table 1). The fact that ‘workers’ have properties that can maximize the fitness of whole colonies (and lack traits improving their fitness within a colony), has been interpreted as the outcome of selection based on fitness differences among colonies [140]. Examples of this include the sterility of workers in eusocial societies, the sacrifice of workers’ lives in defense of the group, and others. But underneath this driving factor of group-level selection there may lie complementary or conflicting selection for individual level benefits. The fact that individuals within the superorganisms are usually highly related (like cells within an organism and cells within a tumor) supports natural selection for group-level traits [141,142]. For example, the cooperative genes of sterile workers are passed on through their fertile sisters in the superorganism, much like the genes of somatic cells are passed on by the germ cells of a multicellular body. Group and organism level selection are therefore intertwined, creating a mosaic of natural selection that shapes these societies [143].

Because sterile workers are analogous to somatic cells, we might appeal to disposable soma theory [144] to predict that the workers would be more susceptible to cancer than the reproductive members of the community. Currently, there is no data to test that hypothesis. However, in almost every social insect society (including ants, bees, wasps and termites) there is a physiological and reproductive division of labor that is also associated with increases in longevity for the reproductive members. Social insect queens do not age gradually. Instead, they die suddenly at an old age [145]. In termites, the disparity in longevity between queens and workers increases with social complexity. The more primitive wood roaches have less of a disparity than their relatives, the advanced eusocial termites, and similar discrepancies are prominent in ant societies [146]. In all of the cases of increased longevity in reproductive members of society, there seems to be hormone mediated pathways with connections to such highly conserved mechanisms as the IIS/TOR signaling systems [147]. This suggests possible connections to research in longevity in many other vertebrates (including in eusocial naked mole rats, who famously have very low rates of cancer) [148,149]. Transposable elements also seem to play a role in senescence in termite castes, with queens and in-nest workers keeping them suppressed longer than out-of-nest (riskier work assignment) workers [150].

While cancer literature regularly borrows concepts from social evolution theory, perhaps social evolution theory might also borrow concepts from cancer. Are there forms of ‘social cancer’ in superorganisms, where organisms within the colony start reproducing out of control, to the detriment of the superorganism? We see high levels of coordination in all eusocial societies and relatively little unsanctioned reproduction by workers [151]. Many eusocial societies show ‘policing’ behaviors where unsanctioned reproductive members are punished or removed in a manner reminiscent of apoptosis or immune surveillance of mutant cells in organisms [152]. For example, in honeybees, the few workers that have developed ovaries are attacked by nestmates, and their eggs are eaten [153]. Thus, most eusocial societies seem remarkably ‘cancer-free’ in this sense. But an exception is found in the Japanese ant *Pristomyrmex punctatus*, a species with no queens. Instead, monomorphic workers reproduce en masse when they are young and become workers as they age. Into this apparently egalitarian social world, a cheater lineage has been described, comprising large bodied female workers with more ovarioles than is typical, who do little useful work other than laying eggs [154]. It may be that on closer examination there are more such instances of what could be seen as cheaters or social cancer in eusocial species.

A noted feature of many cancers is the increased incidence of cancer with age. In many eusocial societies, the senescence or loss of the dominant reproductive member (the queen) leads to a reappearance of previously suppressed independent reproduction. For example, in Formica ants, in colonies who have lost their queen, independent worker reproduction increases [155]. This appearance of more decentralized reproduction resembles cancer in organisms. But it must be emphasized that this non-centralized reproduction in aging or orphaned colonies may be adaptive for eusocial societies. By contrast, it is difficult to suggest that cancer in elderly organisms is ever adaptive for the individual. Still, it does show how group selection can create colony-level adaptations that suppress individual-focused adaptations and how, given a change in circumstances or loss of regulation, the individual-fitness enhancing actors reassert themselves.

Finally, it is arguable that social cancers do occur frequently in another sense: through parasitism. Many eusocial species can be successfully invaded and parasitized by other species. One example of this is the parasitic Cape Honey bee [156], and there are numerous examples within ants of such social parasitism [157]. It has been suggested that very often these parasites are close relatives to the host species, essentially parasitic lineages that are taking advantage of their near-relatives. This is analogous to transmissible cancers, described in various species, for example the canine transmissible venereal tumor [158] or the Tasmanian devil facial tumor disease [159,160], where clones of tumor cells are transmitted between individuals of the same species. The close phylogenetic relationship of a social parasite with its host allows the social parasite or cancerous lineage to ‘crack the code’ of social life (evading the social or individual immune response), whether in an ant colony or a new body. Finally, there are the myriad and fascinating colony parasites (known in ants as myrmecophiles) that invade ant colonies [161]. These parasites are often able to invade the unique ecological niche that is the ant colony, displaying adaptations that allow them to insinuate themselves into colony life and imitate the ants’ chemical and behavioral language. Many similar parasites can be found afflicting both honeybees and termites. Similarly, some transmissible cancers can even parasitize different host species. This has been observed in a transmissible leukemia in mussels [162,163] and in several cases of seemingly cancerous tapeworm cells growing inside human hosts [123].

## Interactions between levels of selection

Price’s formulation of multilevel selection, in which selection on a trait can be decomposed into the separate contributions of selection at the different levels [6,161], requires that the results of selection at both levels are passed onto the next generation [6,164]. Because the results of cellular level selection are generally not passed on to the next generation of the organisms, cancer is not an example of this strong sense of multilevel selection at the cellular and organismal level [164], although there are interesting exceptions in transmissible cancers [165] and gamete-level selection [166].

Are there cases of multilevel selection in Price’s sense among the levels of selection we have analyzed? Yes. There is an extensive literature on multilevel selection in the evolution of genomes, which represent the combined effect of selection on transposable elements that may favor an increase in the number of such elements in the genome [167,168]. However, if they disrupt genes that are necessary for cell survival, cell level selection will remove them, and if they disrupt genes that are necessary for development, reproduction or survival of the organism, organismal level selection will remove them. Cancer cells’ fitness produces an obvious constraint on retrotransposons. Cells with highly active retrotransposons are more likely to be killed by a retrotransposon disrupting a gene necessary for cell survival, or by inducing a viral mimicry response [169,170], than cells with quiescent retrotransposons. But in some cases, the two levels may align, as retrotransposons can increase cell fitness. For example, depending on where they land in the genome, they can lead to over-expression of oncogenes [171] or down-regulation or disruption of tumor suppressor genes [172], a phenomenon called insertional mutagenesis.

Mitochondrial phenotypes may represent the combined action of selection at the mitochondrial, cellular and organismal levels [78,173]. Similarly, ecDNA may evolve features, such as small size, due to selection at the level of ecDNA. But they may also evolve features, such as carrying oncogenes, due to cellular-level selection. Whether or not ecDNAs evolve through organismal-level selection depends on whether the ecDNAs are in germ cells.

Phenotypes of collections of cells, such as crypts and metastases, are the result of interactions between selection on cells and selection on collections of cells. Selection at the cellular level for increased cellular proliferation may either disrupt or enhance the reproduction of epithelial proliferative units. We have already argued that there is interaction between selection at the cellular level, which should select for all cancer cells to act like stem cells, and selection at the level of colonies of stem cells and their non-stem progeny, which may act like proto-multicellular organisms [106].

Selection at the metastasis level for cells to detach and move away, may be detrimental or possibly beneficial at the cellular level. Selection at both the cellular and metastatic level may lead to enhanced cell proliferation and survival. However, the vast majority of cells that leave a tumor do not establish new metastases, so cell migration may often be deleterious at the cellular level [174,175]. It may even be the case that previous work has conflated disease caused by reproduction at two different levels in the case of metastases. Metastatic cancer is generally much more lethal than non-metastatic cancer [176]. Is that because the metastases are dividing out of control or because the cancer cells are dividing out of control?

Conflict between levels of selection is mitigated in most organisms by passing each generation through a single (germ) cell bottleneck. This prevents cancers from being transmitted to the next generation, but it also prevents rogue proliferative units as well as most ecDNA and damaged mitochondria from being passed on to offspring. Similarly, since most superorganisms are established by a single inseminated queen, it is difficult for ‘cheating’ or ‘cancerous’ non-sterile workers to get into the next generation of the colony, unless it evolves a social parasitism strategy.

## Cancer at different levels of organization

**Fig 2:**
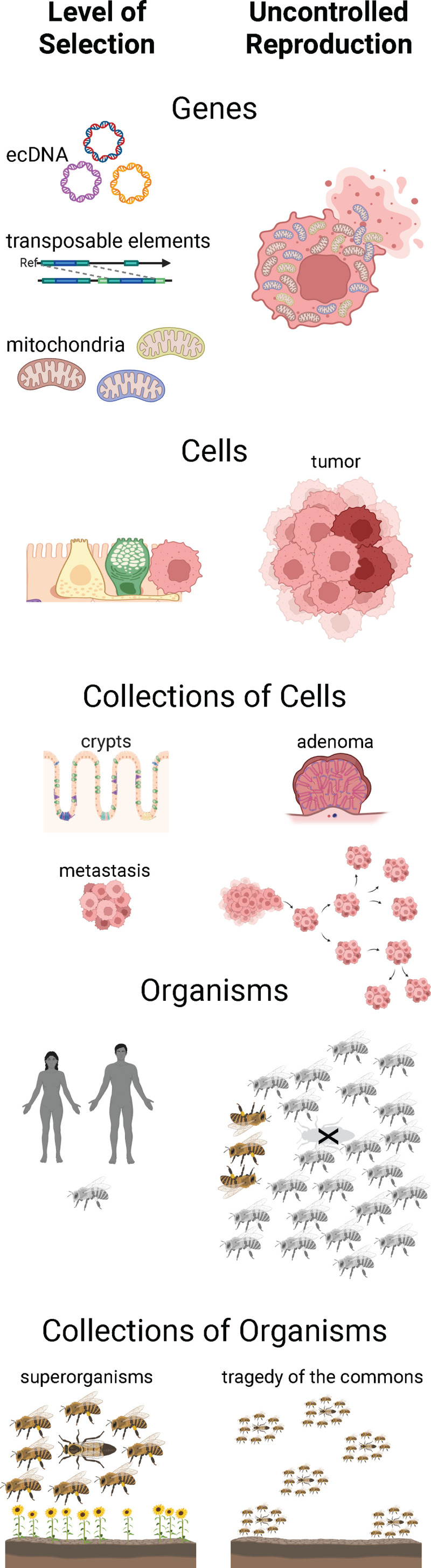
Potential cancer-like phenomena at different levels of selection. If cancer is the uncontrolled reproduction of cells that destroys the higher level of organization, including well-ordered tissues and the host organism, then other levels of selection (on the left) might also reproduce out of control (on the right), destroying higher levels of organization. Selection on retrotransposons, generating highly active retrotransposons, may lead to cell death if they cause to much DNA damages trigger an immune response. Selection on mitochondria might lead to them reproducing out of control, potentially destroying the cell. Selection on cells, leading to uncontrolled proliferation, leads to tumor formation, not only destroying the epithelial proliferative units (and other tissue structures), but often killing the organism with a lethal cancer. If selection on intestinal crypts leads to crypts starting to reproduce out of control, they will likely produce adenomas, which may eventually evolve into cancers. Selection on metastasis for the generation of more metastasis may also destroy the organism. Organismal level selection on workers in a eusocial superorganism would favor workers that regain the ability to reproduce, but by siphoning off resources and disrupting the functioning of the colony, such a mutant worker population would likely destroy the superorganism. If superorganisms (or organisms) reproduce too much, in some cases they destroy their environment and cause their population to crash, in a tragedy of the commons. It is not clear if there has been sufficient selection at those higher levels of organization to prevent such tragedies. Figure created with BioRender, https://BioRender.com.

Careful readers will have noticed that we have discussed two different types of cancer across these levels of organization. We are interested in how selection at different levels of organization affects the disease that we call cancer, and how we might use those insights to improve prevention and management of that disease. In addition, we asked the question, are there cases where uncontrolled reproduction at a lower level of organization destroys the entity at a higher level of organization (Fig 2)? If the disease we call cancer is the uncontrolled reproduction of cells that results in the destruction of the organism, are there analogous phenomena at different levels? This leads to some intriguing questions. Do workers in superorganisms ever start reproducing out of control until they kill the superorganism? Do retrotransposons or mitochondria ever start reproducing out of control until they kill the cell?

The fact that selfish mtDNA mutations can occur raises the theoretical possibility that mitochondrial genomes might start reproducing out of control to the point that they kill their host cell, similar to cancer cells reproducing out of control and killing their host organism (Fig 2). This would be difficult to observe, and there have been no reports of it yet. The closest observation may be in renal oncocytomas, a rare benign tumor that is so packed with mutant mitochondria that the oncocytoma cells are swollen. The excess of mitochondria seem to impair the oncocytoma cells from evolving into cancers [177,178], which suggests a conflict between selection at the mitochondrial and cellular levels.

## Conclusions

Cancer is not simply a case of natural selection on cells resulting in the death of their host. Rather, there are layers upon layers of natural selection in cancer, sometimes at odds with each other, and sometimes amplifying each other. In addition to selection at the level of cells and organisms, mitochondria and superorganisms also meet the necessary and sufficient criteria for natural selection. Whether or not ecDNA, retrotransposons, epithelial proliferative units, clusters of cancer stem cells and their progeny, and micrometastases are additional levels of selection during cancer progression remain open questions, which hinge on whether some of those entities are better at surviving and reproducing than others. While this may appear overwhelming, when we try to treat or prevent cancer, some of these levels of selection may reveal new clinical pathways for improving human health. Can we prevent cancers by inhibiting the reproduction of epithelial units with mutant stem cells? Can we enhance the fitness of retrotransposons in cancer cells to the detriment of the cancer? We are just beginning to appreciate, let alone harness, the many levels of selection in cancer.

## Abbreviations

**:**
ecDNA: extra-chromosomal DNA
mtDNA: mitochondrial DNA
MLS1: multilevel selection 1
MLS2: multilevel selection 2
